# AL amyloidosis with primary presentation of multiple serous cavity effusion and severe cholestasis: a case report and review of literature

**DOI:** 10.1186/s12876-022-02201-4

**Published:** 2022-03-18

**Authors:** Kehui Liu, Yezhou Ding, Yumin Xu, Weiliang Tang, Mingyang Feng, Yunye Liu, Shisan Bao, Hui Wang

**Affiliations:** 1 Department of Infectious Diseases, Ruijin Hospital, Shanghai Jiao Tong University School of Medicine, Shanghai, 200025 China; 2 Department of Infectious Diseases, Ruijin Hospital North, Shanghai Jiao Tong University School of Medicine, Shanghai, 201801 China

**Keywords:** Primary amyloidosis, Multiple serous cavity effusion, Cholestasis, Prognosis, Case report

## Abstract

**Background:**

Immunoglobulin light chain (AL) amyloidosis commonly affects the kidney or heart, but may also involve the liver at a histopathological level. Early diagnosis of AL amyloidosis is important for proper management with desirable outcome. We reported here an unusual case of AL amyloidosis, presenting primarily with multiple serous cavity effusion, accompanied with rapidly progressive cholestasis.

**Case presentation:**

A previously healthy 63-year-old man presented with dysuria, frequent urination, oliguria and oedema of lower extremities for one month, accompanied with jaundice and hypoproteinemia. CT demonstrated multiple serous cavity effusion, focal hypodense lesions in the liver, and focal low-density in the spleen. Laparoscopy with liver biopsy revealed liver and spleen fibrosis with congestion, no visceral rupture, following haemorrhagic ascites from abdominocentesis. This patient was transferred to our (tertiary) hospital. The diagnosis of amyloidosis was confirmed with histopathology/immunohistochemistry. Haematopoietic stem cell transplantation was not applicable, however chemotherapy was advised, due to the patient’s Mayo score 3. The patient declined chemotherapy and was self-discharged back to his hometown hospital with palliative care, however only lasted a further one-month.

**Discussion:**

The lesson we have learnt from this case that any patients with multiple serous cavity effusion and isolated hepatic involvement, primary amyloidosis should be considered. Multiple serous cavity effusion may serve as an indicator for poor prognosis of hepatic AL amyloidosis.

## Background

Immunoglobulin light chain (AL) amyloidosis is due to deposition of protein derived from AL fragments to extracellular tissue [1, 2]. The clinical presentation is protean, depending on affected organs [3]. It is critically important to diagnose AL amyloidosis at the early stage for proper management with desirable outcomes. We reported here an unusual case of AL amyloidosis, presenting primarily with multiple serous cavity effusion accompanied with rapidly progressive cholestasis.

## Case presentation

### History of present illness

A 63-year-old man visited a local hospital, presenting with dysuria, frequent urination, oliguria and lower extremities oedema. The chief complaint was oedema of lower extremities, abdominal distension, nausea and progressive jaundice for one month. No previous abnormal history. On admission, laboratory investigations revealed jaundice (serum total bilirubin, 133 µmol/L) and hypoproteinemia (serum albumin 27 g/L). Computed tomography (CT) revealed multiple serous cavity effusion (pleural, peritoneal and pelvic cavity effusions) and focal hypodense lesions in the liver, and multiple low-density focal lesions in the spleen. Intravenous contrast CT further revealed uneven enhancement without mass effects in the liver and spleen. Initially, Budd Chiari syndrome was considered following magnetic resonance imaging (MRI) examination, revealing local stenosis and near occlusion of the hepatic segment of the inferior vena cava. At the time of admission in the local hospital, haemorrhagic ascites was observed during abdominocentesis. Due to sudden dropping of blood pressure, emergency laparoscopy was performed, revealing liver and spleen fibrosis with congestion, but no obvious visceral rupture. During the operation, liver biopsy was also performed for diagnosis, and abdominal irrigation and drainage were applied. Liver histopathology showed liver fibrosis. Autoimmune liver disease was also considered, but without ruling out of Budd Chiari syndrome. The patient’s condition continued deteriorating with unstable blood pressure and fast progression of jaundice, as well as, large volume ascites. No specific treatment was able to be offer in the local hospital, due to uncertain diagnosis. Therefore, the patient was transferred to a tertiary teaching hospital for a more appropriate management. The discharge information from the local hospital was multiple serous cavity effusions, ascites (bloody), accompanied with liver damage, possible cause of autoimmune liver disease or Budd Chiari syndrome.

### History of past illness

There was no history of fever, asthenia, vomiting, hematemesis, melena, abdominal pain, nor alteration in bowel movement. This patient had right 11th rib fracture, due to blunt injury two months prior to admission. His past medical history was unremarkable, particularly without prior history of liver disease. No history of smoking and drinking.

### Personal and family history

His mother died of liver disease without confirmed diagnosis. He had no any specific personal or family history of other disease.

### Physical examination

Jaundice was obvious at the physical examination. Three abdominal drainage tubes were not clotted. There was mild tenderness and percussion pain in the right upper quadrant abdomen, but no rebound tenderness. Liver was reached 2 cm below the right costal margin with soft edge and mild tenderness. There was oedema of low limbs. No other obvious abnormality was detected.

### Laboratory tests

Laboratory tests were listed in Table 1. The antibodies were negative against mitochondrial (AMA), anti-nuclear (ANA), hepatitis A, B, C, E, HIV, anti-liver or kidney microsome, and anti-smooth muscle, CMV, EBV and T-SPOT.

**Table 1:** Biochemical characteristic

Dates	1 Jan	6 Jan	9 Jan	13 Jan	16 Jan
WBC (3.97–9.15 × 10^9^/L)	11.47	11.5	12.1	12.8	11.6
RBC (4.09–5.74 × 10^12^/L)	5.02	4.98	5.32	5.41	5.5
Hb (131–172 g/L)	159	158	165	174	173
Plt (85–303 × 10^9^/L)	325	356	302	277	230
PT (10–16 s)	13.8	14.1	16.3	16.4	17
INR	1.18	1.21	1.41	1.42	1.48
CRP (< 5 mg/L)	4.1	4.6	3.8	3.6	3.3
PCT (< 10 ng/mL)	0.76	0.97	1.01	1.17	1.24
ALT (10–64 U/L)	82	82	82	80	63
AST (8–40 U/L)	73	91	86	94	64
AKP (38–126 U/L)	566	666	735	774	684
r-GT (7–64 U/L)	513	484	452	410	318
TB (4.7–24 µmol/L)	264.2	405.2	528.2	621.6	637
DB (0–6.8 µmol/L)	142	205	251.9	285.6	291.7
Alb (35–55 g/L)	23	26	26	26	25
Bun (2.5–7.1 mmol/L)	9.6	7.8	7.7	11.2	14.2
Cr (62–115 µmol/L)	74	78	90	117	164

Urinalysis revealed that protein-to-creatinine ratio was 72.09 mg/mmol. IgG was 5.43 g/L (8.6–17.4 g/L), κ free light chain 24.2 mg/L (3.3–19.4 mg/L) and λ free light chain 928 mg/L (5.71–26.3 mg/L), as well as, 1294 mg/24 h for protein (reference range negative, 0 g/L). Serum electrophoresis was negative for Ig, monoclonal immunoglobulin (mAb) kappa and lambda light chains. Urine immunofixation showed the deposition of monoclonal free λ light chains. Ultrasound cardiogram demonstrated that contraction activities of left ventricular wall were slightly less than normal and a small amount of pericardial effusion.

The ascites was yellow and turbid, with negative for Rivalta test. Cell count of the ascites was 15 × 10^6^/L, LDH was 42.4 U/L, protein was 4.40 g/L, and adenosine deaminase was 11.6 U/L.

### Imaging examinations

**Fig. 1:**
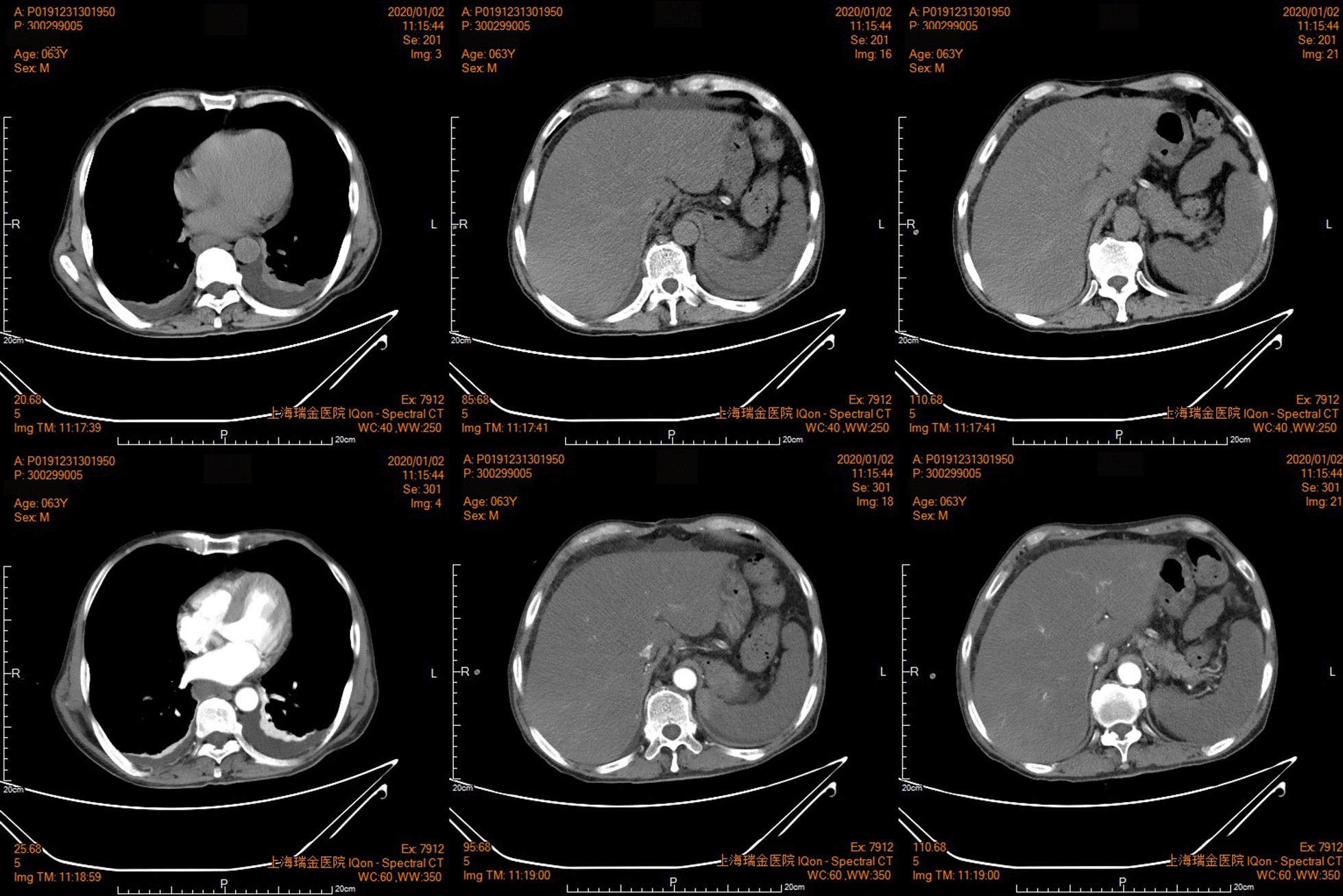
The CT image presenting abdominal pleural effusion, hepatomegaly and splenic infarction

Abdominal ultrasonography revealed multiple serous cavity effusion with hepatomegaly without focal liver lesions. No abnormality was detected in intra- and extrahepatic bile ducts. Splenomegaly was also observed (13.4 cm). The second CT in the referred tertial hospital (Ruijin Hospital) confirmed that there was hepatomegaly, splenic infarction, pleural effusion (Fig. 1) and pericardial effusion, abdominal cavity drainage, oesophageal gastric varices, but no obvious abnormality in portal vein.

### Histopathology and immunohistochemistry

**Fig. 2:**
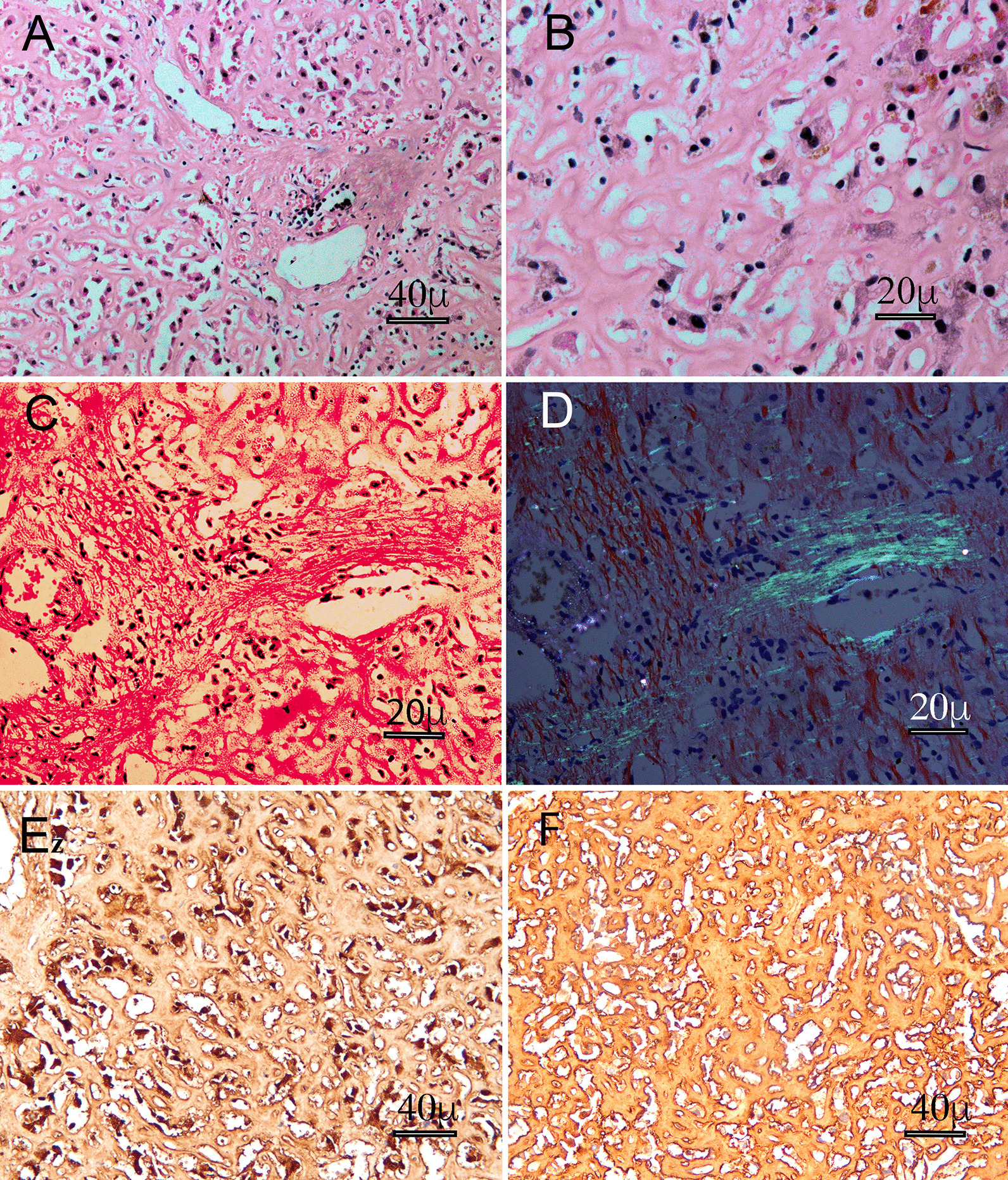
Representative images of hepatic AL amyloidosis of HE staining, showing perisinusoidal pattern of deposition (×200) (**A**); (×400) (**B**); Congo red stain (×400) (**C**) and Congo red apple-green birefringence under polarized light (×400) (**D**); Immunohistochemistry on the liver biopsy with κ chain staining (×200) (**E**) or λ chain staining (×200) (**F**)

To confirm the diagnosis, a well-recognised independent senior pathologist was consulted. The hepatic lobular structure was preserved, and a large amount of amorphous (amyloid) material was diffused in the hepatic sinuses and squeezed the hepatic cord (Fig. 2A, × 200, Leica DM2500 microscope, objective HI Plan × 20; B, × 400, Leica DM2500 microscope, objective HI Plan × 40). Importantly, apple-green birefringence was observed under polarized light (Fig. 2D, × 400, Olympus BX53 microscope, objective UPlan × 40) with Congo red stained (Fig. 2C, × 400, Olympus BX53 microscope, objective UPlan × 40). Immunohistochemistry on the liver biopsy revealed that κ chain was heavily deposited in the sinusoid of the liver (Fig. 2E, × 200, Leica DM2500 microscope, objective HI Plan × 20); whereas λ chain was more diffused distributed in the liver (Fig. 2F, × 200, Leica DM2500 microscope, objective HI Plan × 20). Thus, primary liver AL-Amyloidosis was confirmed, in conjunction with the histopathology and Congo red staining.

Following the diagnosis of hepatic amyloidosis, the indexes of the patient's liver, kidney and heart deteriorated sharply, while prothrombin time was prolonged dramatically. The patient and his family refused further invasive examination. Thus, no bone marrow examination was permitted.

### Final diagnosis

This patient was diagnosed with primary hepatic amyloidosis, based on the final pathologist report.

### Outcome and follow-up

The most critical issue in hepatic amyloidosis is early diagnosis, because any delayed confirmation could cause extremely serious consequence. The condition of this patient, including liver, heart and renal, deteriorating extremely rapidly (Table 1), showing estimated glomerular filtration rate (eGFR) was 48 mL/min/1.73 m^2^; NT-pro BNP, 2976 pg/mL; cardiac troponins, 0.18 ng/mL, and serum free light chain difference (DFLC) was 903.8 (≥ 180) mg/L. Thus, Mayo score 3 for AL amyloidosis was given to this patient [4]. Upon the diagnosis, a haematologist was consulted, and hematopoietic stem cell transplantation was not advised for this patient, however chemotherapy was recommended. This patient declined chemotherapy and elected to be self-discharged back to his hometown for palliative care only. The patient died of multi-organ failure one month after discharge.

## Discussion and conclusions

We reported here a rare case of hepatic primary amyloidosis, presenting with multiple serous cavity effusions and liver damage, as the primary manifestations. Due to lack of specific clinical manifestations and auxiliary examinations, the diagnosis was not established even after first liver biopsy in the local hospital of hometown. The diagnosis was finally confirmed by second histopathology, using histopathology/immunohistochemistry.

The extremely unusual primary symptoms and signs in this case were multiple serous cavity effusions and liver damage. The most common clinical presentations in amyloidosis are asthenia and dyspnoea [2]. This patient presented with non-classical clinical presentation, including abdominal distension, low limbs oedema and oliguria. There was upregulated bilirubin (mainly conjugated) with relative normal renal and cardiac functions and PT. It was a big challenge to the clinicians that multiple serous cavity effusions and bloody ascites within such short period, especially there was no space occupying lesion, although exploratory laparoscopy with liver biopsy was performed.

A tentative diagnosis was Budd Chiari syndrome or hepatic sinusoidal obstruction syndrome by the local hospital. Finally, the diagnosis of amyloidosis was suggested by the multi-disciplinary team in our (tertiary) hospital and was confirmed by the secondary opinion from a pathologist, in conjunction of immunohistochemistry. There are a few reports of cases of Budd-Chiari syndrome related of AL-Amyloidosis [5, 6]. However, this patient was eliminated from Budd-Chiari syndrome after the enhanced CT examination. The delayed diagnosis for primary amyloidosis for this patient was mainly due to lack of specific presentations, which are highly dependent on the extent and the organ(s) [4, 7]. We realise that early diagnosis of primary amyloidosis is critical for the possible outcomes of management. Our finding is in line with a review of 98 patients with hepatic amyloidosis, recommending that primary amyloidosis should be considered for the patients with hepatomegaly and elevation of alkaline phosphatase [8]. In addition, our data is consistent with others, demonstrating that hyperbilirubinemia and marked elevation of serum alkaline phosphatase are poor prognostic factors of hepatic amyloidosis [9].

Chemotherapy and/or autologous stem cell transplantation probably is the first choice for the management of amyloidosis [4], depending on the stage and suitability [10]. In view of progressive liver, renal and cardiac damages, stem cell transplantation for this patient was deemed inappropriate, due to a poor prognosis (estimated less than 6 weeks).

There is a case report of primary hepatic amyloidosis [11], presenting hepatomegaly and abdominal pain without positive for other viral and/or autoimmune markers. It is confirmed that primary hepatic amyloidosis, using polarizing microscopy with Congo red staining of apple green birefringence. In line with Rathi’s report, our current case was also finally diagnosed, using polarizing microscopy. The uniqueness of the current case was due to the clinical presentation of multiple serous cavity effusion with severe cholestasis, which was confused with autoimmune liver disease [12] and Budd Chiari syndrome [13]. We understand that multiple serous cavity effusions and liver damage are quite common at the terminal stage of systemic AL amyloidosis. However, this patient presented with multiple serous cavity effusions and liver damage at the rather early stage with jaundice, and the chief complain was urological related symptoms when he was visiting the local hospital. This case provided relevant information for the clinicians to consider for differential diagnosis of potential systemic AL amyloidosis. The lesson we have learnt from this case is that in patients with multiple serous cavity effusions and isolated hepatic involvement, liver biopsy must be performed and polarizing microscopy with Congo red and immunohistochemical of kappa/lambda staining must also be utilised for differential diagnosis. Delayed diagnosis of primary hepatic amyloidosis may have serious consequence, i.e. missing out the possibility of more effective stem cell transplantation [4] in combination of chemotherapy [4] with desirable outcomes. Finally, we also conclude from this rare case that multiple serous cavity effusions may be an indicator for poor prognosis of hepatic amyloidosis.

## Data Availability

All data from manuscript will be available upon request by contacting Dr. Hui Wang.

## Abbreviations

AKP: Alkaline phosphatase
AL amyloidosis: Immunoglobulin light chain amyloidosis
Alb: Serum albumin
ALT: Alanine aminotransferase
AMA: Against mitochondrial
ANA: Antinuclear
AST: Aspartate aminotransferase
Bun: Blood urea nitrogen
CMV: Cytomegalovirus
Cr: Serum creatinine
CRP: C-reactive protein
CT: Computed tomography
DB: Direct bilirubin
DFLC: Serum free light chain difference
EBV: EB virus
eGFR: Estimated Glomerular filtration rate
Hb: Haemoglobin
HIV: Human immunodeficiency virus
INR: International normalized ratio
LDH: Lactate dehydrogenase
mAb: Monoclonal immunoglobulin
MRI: Magnetic resonance imaging
PCT: Procalcitonin
Plt: Platelet
PT: Prothrombin time
γ-GT: γ-Glutamyl transpeptidase
RBC: Red blood count
TBiL: Total bilirubin
T-SPOT: T-SPOT.TB
WBC: Leukocyte count
